# Three-year progression-free survival of a patient with concomitant mucinous adenocarcinoma of the colon with peritoneal dissemination and multiple myeloma who received lenalidomide: a case report

**DOI:** 10.1186/s40792-024-01838-5

**Published:** 2024-02-07

**Authors:** Koki Tamai, Hajime Hirose, Yo Akazawa, Yukihiro Yoshikawa, Masatoshi Nomura, Hiroshi Takeyama, Masahiro Tokunaga, Mitsuyoshi Tei, Shu Okamura, Yusuke Akamaru

**Affiliations:** 1 Department of Gastroenterological Surgery, Osaka Rosai Hospital, 1179-3 Nagasone-Kitaku, Sakai, Osaka 591-8025 Japan; 2 Department of Surgery, Suita Municipal Hospital, Kishibeshinmachi 5-7, Suita City, Osaka 564-8567 Japan; 3 Department of Hematology, Suita Municipal Hospital, Kishibeshinmachi 5-7, Suita City, Osaka 564-8567 Japan

**Keywords:** Lenalidomide, Mucinous adenocarcinoma, Peritoneal dissemination, Multiple myeloma

## Abstract

**Background:**

Concomitant multiple myeloma (MM) and other primary malignancies is rare. Therefore, the treatment outcomes of patients with these conditions have not been well discussed. Lenalidomide is an oral thalidomide analog drug used for MM. Recently, the antitumor effect of lenalidomide has been gaining attention, and lenalidomide has been applied for managing solid tumors. The current case showed the treatment course of a patient treated with lenalidomide for concomitant MM and colon cancer with peritoneal dissemination.

**Case presentation:**

A 74-year-old female patient receiving treatment for MM was diagnosed with mucinous adenocarcinoma of the transverse colon. The patient was clinically diagnosed with stage IIIC T4aN2M0 disease. Subsequently, laparoscopic colectomy with lymph node dissection was planned. However, intraperitoneal observation revealed peritoneal dissemination that had sporadically and widely spread. Therefore, palliative partial colectomy was performed to prevent future hemorrhage or obstruction. The patient was discharged on the 10th postoperative day without postoperative complication. Based on the patient’s preference, lenalidomide was continually administered for MM without systemic chemotherapy. The patient survived for > 36 months without any signs of tumor progression.

**Conclusion:**

The current case first showed the treatment course of concomitant MM and colon cancer. The antitumor effect of lenalidomide can possibly contribute to 3-year progression-free survival in patients with mucinous adenocarcinoma of the colon with peritoneal dissemination.

## Background

Multiple myeloma (MM) is a hematologic malignancy in which cancerous plasma cells accumulate in the bone marrow. Concomitant MM and other primary malignancies are rare. However, patients with MM occasionally develop second primary malignancies (SPM) [1, 2]. However, due to its rarity, the treatment course of patients with these conditions has not been well discussed.

Lenalidomide is an oral thalidomide analog drug used for MM, and it has immunomodulatory and antiangiogenetic effects. Due to its antitumor effect, several attempts have been made recently to treat solid tumors with lenalidomide alone or in combination with chemotherapy [3].

Herein, we report a case of concomitant MM and mucinous adenocarcinoma of the colon with peritoneal dissemination in a patient with 3-year progression-free survival after lenalidomide treatment without systemic chemotherapy.

## Case presentation

**Fig. 1:**
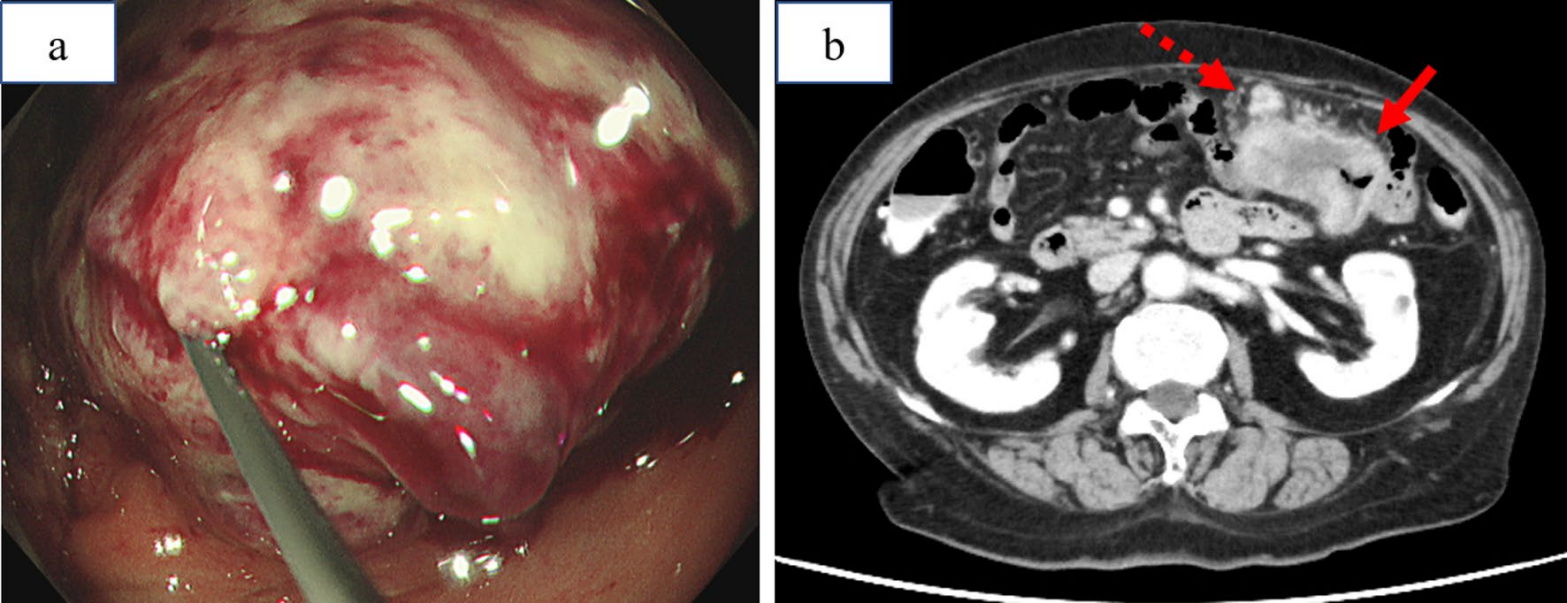
Preoperative examinations. **a** Colonoscopy confirmed an obstructive tumor in the transverse colon. **b** Computed tomography scan showed thickening of the transverse colon (solid arrow) with enlarged lymph nodes (dashed arrow)

A 74-year-old female patient presented with discomfort due to upper abdominal pain. She was diagnosed with MM and was treated with lenalidomide (15 mg/day) for 3 years before visiting the institution. Colonoscopic examination revealed a tumor in the transverse colon, which could not pass through the endoscope (Fig. 1a). Based on the biopsy results, the tumor was diagnosed as mucinous adenocarcinoma. Computed tomography (CT) scan of the abdomen showed irregular circumferential thickening of the transverse colon with enlarged lymph nodes. However, it could not detect distant metastasis (Fig. 1b). Biochemical analysis revealed a high level of carcinoembryonic antigen at 7.9 (normal range: < 5.0) ng/mL and carbohydrate antigen 19-9 at 6372.9 (normal range: < 37.0) U/mL. The patient was clinically diagnosed with stage IIIC T4aN2M0 disease (TNM classification, 8th version).

**Fig. 2:**
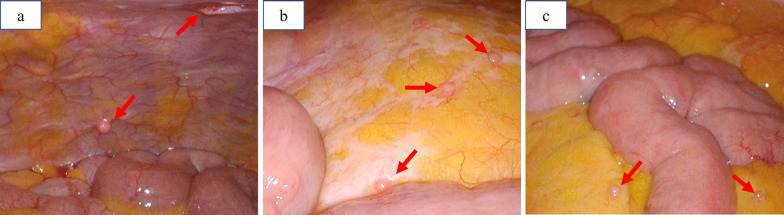
Intraperitoneal surgical view. Small nodules were sporadically and widely spread in the abdominal cavity. **a** Left lateral abdomen. **b** Descending mesentery. **c** Small mesentery

Laparoscopic colectomy with lymph node dissection was planned 20 days after halting lenalidomide. However, intraperitoneal observation revealed that the nodules strongly suspected of peritoneal dissemination were sporadically and widely spread in the abdominal cavity (small mesentery, descending mesentery, and bilateral upper abdominal wall) (Fig. 2). Due to peritoneal dissemination, radical surgery was not suitable. Then, palliative laparoscopic partial colectomy without sufficient lymph node dissection was conducted to prevent future hemorrhage or obstruction. The surgical duration was 207 min, and the volume of blood loss was 10 mL.

**Fig. 3:**
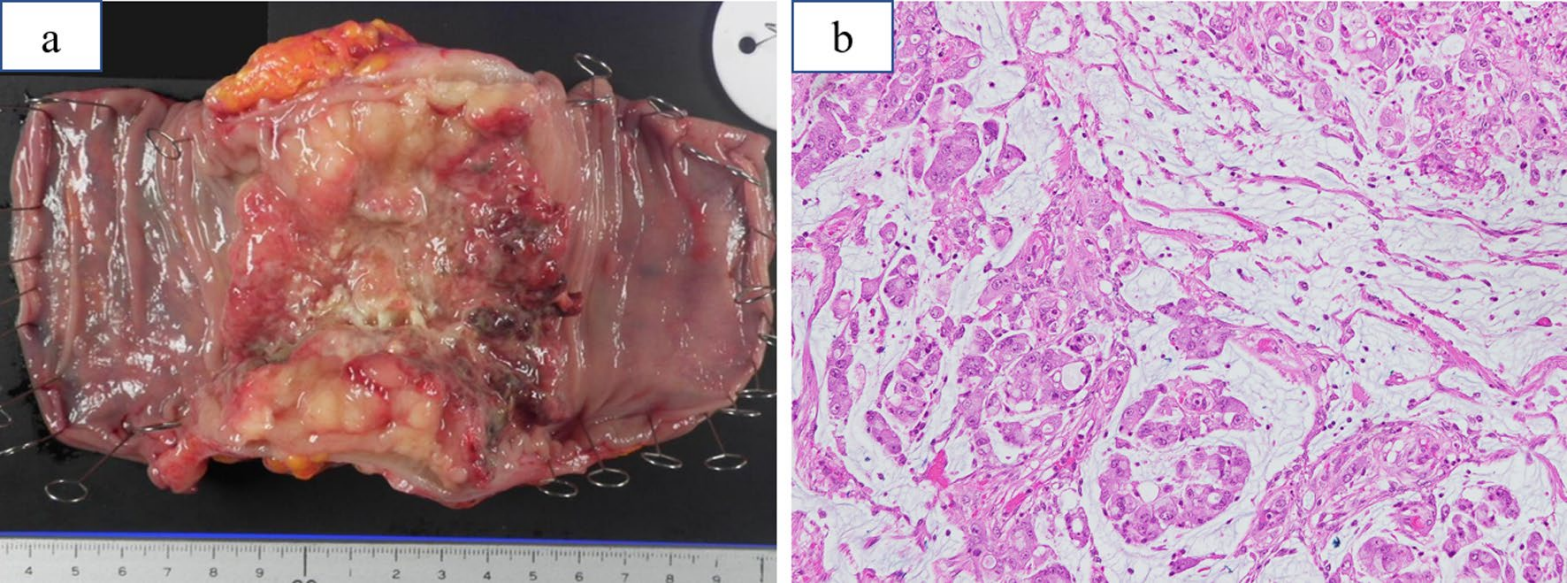
The resected specimen. **a** Surgical specimen revealed an 80-mm-long circumferential tumor. **b** Histopathological examination revealed that the intra-abdominal nodules were mucinous and poorly differentiated adenocarcinoma (hematoxylin and eosin staining, × 200)

Histopathological examination confirmed the diagnosis of mucinous adenocarcinoma with serosal erosion, and one positive lymph node metastasis out of four resected lymph nodes. Moreover, an intraperitoneal nodule, resected intraoperatively, was pathologically diagnosed with peritoneal dissemination (Fig. 3). The final diagnosis was stage IVC (T4aN1aM1c). Histologically, after lenalidomide administration, therapeutic effect was not identified. The patient was discharged on the 10th postoperative day without postoperative complication. Although peritoneal dissemination still remained, the patient refused chemotherapy for colon cancer due to fear of adverse events. She decided to receive treatment with lenalidomide only under the management of the department of hematology. Subsequently, administration of lenalidomide was resumed 30 days after surgery. She underwent outpatient follow-up with blood tests, including carcinoembryonic antigen (CEA) and carbohydrate antigen 19-9 (CA19-9) levels test every 3 months and chest and abdominal CT scan every 6 months. She did not present with any signs of tumor progression for > 36 months on regular follow-up (Table 1).

**Table 1:** Changes in tumor marker levels before and 3 years after surgery

	Before surgery	Time after surgery (months)												
		1	3	6	9	12	15	18	21	24	27	30	33	36
CEA level (ng/mL)	7.9	2.1	< 1.7	2.3	1.8	< 1.7	< 1.7	< 1.7	< 1.7	< 1.7	< 1.7	< 1.7	< 1.7	< 1.7
CA19-9 level (U/mL)	6373	223	2.3	< 2.1	< 2.1	4.7	< 2.1	< 2.1	< 2.1	< 2.1	< 2.1	< 2.1	< 2.1	< 2.1

*CEA* carcinoembryonic antigen, *CA19-9* carbohydrate antigen 19-9

## Discussion

Mucinous adenocarcinoma is a unique subtype of colorectal cancer, and it accounts for 5%–15% of all colorectal cancer cases [4]. The characteristics of mucinous adenocarcinoma include easy filtration and metastasis and chemo- or radiotherapy resistance [5]. Therefore, the tumor is often more advanced upon diagnosis compared with differentiated adenocarcinoma and can be refractory to treatment [6]. In the current case, the patient had peritoneal dissemination, which is associated with a poor survival compared with other metastatic sites. Despite the development of systemic chemotherapy, the prognosis of unresectable peritoneal dissemination remains poor, with a median survival of 11.0–17.9 months [7–9]. Generally, the patient had an unfavorable outcome because of two concomitant conditions (mucinous adenocarcinoma and peritoneal dissemination) with a poor prognosis. However, the patient survived for > 36 months without any sings of tumor progression without systemic chemotherapy. Thus, lenalidomide could have contributed to 3-year progression-free survival.

Lenalidomide is a thalidomide derivative that was approved by the U. S. Food and Drug Administration for treating MM and myelodysplastic syndrome. Lenalidomide has immunomodulatory and antiangiogenetic effects. Lenalidomide, an immunomodulator, inhibits the expression of proinflammatory cytokines [10], reduces the expression of regulatory T-cells [11], stimulates the expression of T-cells inducing cytokine secretion, and promotes natural killer cell cytotoxicity [3]. In addition to these indirect antitumor effects, the antiangiogenetic effect of lenalidomide is suppressing the expression of vascular endothelial factor and basic fibroblast growth factor [12]. Furthermore, lenalidomide induces cell cycle arrest in the G0–G1 phase and apoptosis [13]. Based on these antitumor mechanisms, clinical trials have revealed the effect of lenalidomide monotherapy or combined chemotherapy and antibody therapy against solid tumors, including thyroid, hepatocellular, prostate, lung, and renal cell cancer [3]. In colorectal cancer, the antitumor effect of lenalidomide was revealed in vitro and in vivo [14, 15]. Recent clinical trials have shown that lenalidomide can have antitumor effects [16–18]. However, all these clinical trials were designed to combine lenalidomide with other drugs, such as cytotoxic and antibody drugs, anticipating synergy effects. To the best of our knowledge, this is the first report showing the antitumor effect of lenalidomide monotherapy against colon cancer in clinical settings.

Recently, patients with MM have a high incidence of SPM [1, 2]. Although the pathogenesis of SPM is unclear, the use of alkylating agents, biological factors, genetic predisposition, and immune dysfunctions are a possible mechanism for the development of SPM in patients with MM [1, 19]. Further, growing evidence has revealed that the use of lenalidomide is associated with an increased risk of SPM in MM [20]. Increased surveillance and genetic polymorphisms can explain the increased risk of SPM [20, 21]. Lenalidomide is associated with an increased risk of SPM in patients with MM, with a risk ratio of 1.30 [20]. The incidence of SPM ranges from 6.9% to 18.9% [20, 22, 23]. There are only a few reports on the site of occurrence of SPM in patients with MM treated with lenalidomide. Jackson et al. reported that 4 (6.7%) of 60 solid SPMs were diagnosed as colon cancers [23].

As the current case did not have colonoscopy report, it was unclear whether the colon cancer manifested before the onset of MM or developed during MM treatment. If colon cancer was already present upon MM diagnosis, lenalidomide could be a tumor suppressor; thus, it can slow tumor progression. By contrast, if lenalidomide was associated with the development of SPM, whether it is effective in suppressing tumor progression immediately after carcinogenesis or it acts as a tumor suppressor at some point remains unclear. In any case, 3-year progression-free survival was achieved after colectomy. This may be either because the efficacy of lenalidomide was facilitated by reducing tumor burden or because the oncological properties of dissemination, which differed from the primary site, were favorable for lenalidomide. Considering that no apparent therapeutic effect was identified from lenalidomide administration, it may not have a cytocidal effect against the tumor, rather the drug suppressed tumor growth. Zuo et al. reported that first-line therapy with bortezomib and the maintenance use of lenalidomide after lung tumor resection complicated by MM could have helped achieve a satisfactory therapeutic effect for > 3 years without progression despite relapse after the first year [1]. By contrast, Ujiie et al. presented a case with concurrent MM and gastric cancer with rapid liver metastasis progression and subsequent death post gastrectomy and lenalidomide discontinuation [24]. They believed that lenalidomide discontinuation promoted tumor angiogenesis and lowered antitumor immunity. Based on these reports, the continuation of lenalidomide, as in the current case, can be beneficial for tumor suppression.

In the current case, the patient with mucinous adenocarcinoma with peritoneal dissemination survived > 36 months without using systemic chemotherapy. However, the effect and mechanism of lenalidomide against advanced colon cancer remains unclear. Although preclinical and clinical research has validated the antitumor effect of lenalidomide, data accumulation and research on the benefits of lenalidomide are warranted.

## Conclusion

Herein, we report the first case of mucinous colon cancer with peritoneal dissemination in a patient with MM treated with lenalidomide. Based on our result, lenalidomide may have contributed to 3-year progression-free survival.

## Data Availability

The datasets used and/or analyzed during the current study are available from the corresponding author on reasonable request.

## Abbreviations

CA19-9: Carbohydrate antigen 19-9
CEA: Carcinoembryonic antigen
CT: Computed tomography
MM: Multiple myeloma
SPM: Second primary malignancies
